# Editorial: Physiology and pathophysiology of placenta

**DOI:** 10.3389/fendo.2026.1829406

**Published:** 2026-04-10

**Authors:** Federica Piani, Giovanni Tossetta

**Affiliations:** 1 Division of Physiology and Pathophysiology, Otto Loewi Research Center for Vascular Biology, Immunology and Inflammation, Medical University of Graz, Graz, Austria; 2 Sex and Gender in Disease Pathophysiology Research Unit, Medical University of Graz, Graz, Austria; 3 Department of Human Sciences and Promotion of the Quality of Life, San Raffaele Open University, Rome, Italy

**Keywords:** pathway, placenta, pregnancy, pregnancy complication, treatment

## Introduction

The modern understanding of the human placenta has evolved from viewing it as a simple transient barrier to recognizing it as a highly sophisticated, dynamic organ that dictates long-term health outcomes for both mother and child. Recent advances in placental biology are beginning to unravel the molecular and cellular mechanisms underlying these processes, revealing how disturbances in placental signaling, including hypoxia- and angiogenesis -related pathways, may contribute to a wide spectrum of pregnancy complications (1, 2).

## Molecular underpinnings and cellular potential

At the core of placental development are trophoblast cells, whose migration and invasion are regulated by complex signaling cascades. Pathways such as Wnt, Notch, and TGF-beta are essential for branching morphogenesis (3), while dysregulation in these signals, including downstream inflammatory mediators and oxidative stress–related pathways, are linked to conditions like preeclampsia (PE) (4, 5). Beyond native development, regenerative medicine is exploring the versatility of human amniotic membrane mesenchymal stem cells (hAmMSCs) (6). These cells exhibit the potential to differentiate into steroidogenic lineages when induced by factors like SF-1 and WT1, offering hope for treating endocrine disorders.

## The immunological and microbial landscape

One of the most revolutionary shifts in reproductive science is the discovery of the placental microbiota (7). Advances in high-throughput sequencing have challenged the “sterile womb” dogma, suggesting that low-level microbial communities, potentially originating from maternal oral or gut sites, play a role in local homeostasis. Imbalances in these communities are increasingly associated with preterm birth and fetal growth restriction (FGR). Complementing this environment are Hofbauer cells, the placenta’s specialized macrophages, which act as primary immune responders to infections (8). Understanding their unique polarization and function is critical for decoding maternal-fetal immune dialogues.

## Endocrine dynamics and metabolic regulation

The placenta serves as a primary endocrine hub, secreting essential peptide hormones such as hCG and hPL that coordinate pregnancy (9). This hormonal activity is supported by sophisticated vesicular trafficking and secretion pathways within placental cells. Metabolic proteins also emerge as vital biomarkers of fetal well-being; for instance, the Klotho protein is significantly reduced in the placentas and blood of women with intrauterine growth restriction (IUGR) (10). This reduction likely influences fetal development through the GH/IGF-1 axis. Furthermore, in cases of gestational diabetes mellitus (GDM), circulating levels of GPIHBP1, a protein essential for triglyceride lipolysis, show significant correlations with neonatal birth weight and oxygenation, highlighting its role in maternal-fetal lipid metabolism (11).

## Navigating high-risk pathologies

Clinical management of placental disorders remains a high-stakes endeavor. Placenta accreta spectrum represents a severe threat where abnormal placental invasion leads to life-threatening hemorrhages. Identifying risk factors for emergency cesarean sections, such as a history of previous cesareans and low preoperative hemoglobin, is crucial for improving outcomes (12). Additionally, decreased maternal plasma levels of APOA1 have been identified as a significant predictor of placenta accreta severity and the necessity for blood transfusions or hysterectomies (13).

Maternal systemic conditions also leave a mark on the placenta; hypothyroidism during pregnancy is associated with distinct pathological features, including insufficient vascular perfusion and increased chronic inflammation (14). Rarer conditions, such as intraplacental choriocarcinoma, must be monitored through vigilant beta-hCG surveillance, as they can lead to spontaneous, massive fetomaternal hemorrhage with high perinatal mortality (15).

## Protective strategies and technological innovation

The quest for protective agents has identified Pyrroloquinoline quinone (PQQ) as a promising nutrient. In experimental models, PQQ pre-conditioning has successfully alleviated inflammation-induced placental damage and improved fetal survival rates (16). Such protective systems are vital because placental health directly impacts the neonatal brain; dysregulation of the placental serotonergic system is now clearly linked to early impairments in infant neurodevelopmental scores (17).

Finally, for the most vulnerable extremely preterm infants requiring extracorporeal life support, technology must account for neonatal biological specificities. Research has demonstrated that neonatal blood exhibits significantly higher susceptibility to hemolysis compared to adult human or standard porcine blood models (18). This finding necessitates the development of specialized miniaturized devices, such as artificial placentas, tailored specifically to the fragile nature of neonatal erythrocytes.

Taken together, the studies included in this Research Topic underscore the remarkable complexity of the placenta as an endocrine, metabolic, immunological, and vascular organ that lies at the center of maternal-fetal health. By integrating insights from molecular biology, experimental models, clinical cohorts, and translational research, this Research Topic highlights the multifaceted nature of placental function and its critical role in determining pregnancy outcomes and early life development. Continued interdisciplinary research in placental biology will be essential for improving the diagnosis, prevention, and treatment of pregnancy-related disorders and for advancing our understanding of how early life environments shape health across the lifespan.

## References

[1] Fantone S Ermini L Piani F Di Simone N Barbaro G Giannubilo SR . Downregulation of argininosuccinate synthase 1 (ASS1) is associated with hypoxia in placental development. Hum Cell. (2023) 36:1190–8. doi: 10.1007/s13577-023-00901-x. PMID: 36995581

[2] Piani F Tossetta G Fantone S Agostinis C Di Simone N Mandalà M . First trimester CD93 as a novel marker of preeclampsia and its complications: A pilot study. High Blood Press Cardiovasc Prev. (2023) 30:591–4. doi: 10.1007/s40292-023-00608-y. PMID: 38010536

[3] Liu L Tang L Chen S Zheng L Ma X . Decoding the molecular pathways governing trophoblast migration and placental development; a literature review. Front Endocrinol (Lausanne). (2024) 15:1486608. 39665023 10.3389/fendo.2024.1486608 PMC11631628

[4] Marzioni D Piani F Di Simone N Giannubilo SR Ciavattini A Tossetta G . Importance of STAT3 signaling in preeclampsia (Review). Int J Mol Med. (2025) 55(4). 10.3892/ijmm.2025.5499 PMC11878484 39918020

[5] Annesi L Tossetta G Borghi C Piani F . The Role of Xanthine Oxidase in Pregnancy Complications: A Systematic Review. Antioxidants (Basel). (2024) 13(10). 10.3390/antiox13101234 PMC11505381 39456486

[6] Miyazaki Y Orisaka M Fujita Y Mizutani T Yazawa T Yoshida Y . Steroidogenic differentiation of human amniotic membrane-derived mesenchymal stem cells into a progesterone-/androgen-producing cell lineage by SF-1 and an estrogen-producing cell lineage by WT1-KTS. Front Endocrinol (Lausanne). (2024) 15:1410433. 39359415 10.3389/fendo.2024.1410433 PMC11445051

[7] Xie Z Chen Z Chai Y Yao W Ma G . Unveiling the placental bacterial microbiota: implications for maternal and infant health. Front Physiol. (2025) 16:1544216. 40161970 10.3389/fphys.2025.1544216 PMC11949977

[8] Wakoli DM Ondigo BN . Isolation and characterization of Hofbauer cells from human term placentas. Front. Immunol. (2026) 17:1671127.10.3389/fimmu.2026.1671127 41918734 10.3389/fimmu.2026.1671127 PMC13033568

[9] Ahmadi SM Perez ML Guardia CM . Secretion of placental peptide hormones: functions and trafficking. Front Endocrinol (Lausanne). (2025) 16:1584303. 40575259 10.3389/fendo.2025.1584303 PMC12197938

[10] Zeng G Lian J Shen J Shi Y . Expression and clinical significance of Klotho protein in serum, umbilical cord blood, and placenta of pregnant women with intrauterine growth restriction. Front Pediatr. (2025) 13:1611877. 40831795 10.3389/fped.2025.1611877 PMC12358447

[11] Watanabe M Eguchi J Kurooka N Eto E Masuyama H Wada J . Maternal circulating GPIHBP1 levels and neonatal outcomes in patients with gestational diabetes mellitus: a pilot study. Front Clin Diabetes Healthc. (2025) 6:1682012. 41142641 10.3389/fcdhc.2025.1682012 PMC12549623

[12] Li M Zhang H Song X Li L Meng F Chu R . Risk factors for emergency cesarean delivery in patients with placenta accreta spectrum: a multicenter retrospective cohort study. Front Med (Lausanne). (2025) 12:1539998. 40969803 10.3389/fmed.2025.1539998 PMC12440873

[13] Zeng S Qu J Jiang H Shi H Yan J Zhao Y . Decreased plasma APOA1 levels are associated with increased severity of placenta accreta spectrum disorders: a nested case-control study. Front Endocrinol (Lausanne). (2025) 16:1627377. 40778280 10.3389/fendo.2025.1627377 PMC12328184

[14] Bao Z Li Y Xue T Liu Y Yan J Wang X . Pathological features of the placenta in hypothyroidism during pregnancy: a population-based retrospective cohort study in Chinese population. Front Endocrinol (Lausanne). (2025) 16:1671641. 41282300 10.3389/fendo.2025.1671641 PMC12631384

[15] Wang L Li X Bai D Du R Lu L Li Y . Intraplacental choriocarcinoma and spontaneous fetomaternal hemorrhage: Uncovering diagnostic clues in a challenging case. Front Oncol. (2025) 15:1600200. 40958863 10.3389/fonc.2025.1600200 PMC12433862

[16] Han Y Zhu Y Lu X Fan T Yin Y Liu P . Pre-conditioning with PQQ during pregnancy alleviates LPS-induced placental damage and improves the fetal survival and growth in mice. Front Endocrinol (Lausanne). (2025) 16:1617026. 40708725 10.3389/fendo.2025.1617026 PMC12286791

[17] Canul-Euan AA Flores-Pliego A Solis-Paredes JM Espejel-Nuñez A Parra-Hernández SB Mendoza-Hernández T . Placental serotonergic system dysregulation is associated with early impairments in infant neurodevelopment. Front Endocrinol (Lausanne). (2025) 16:1694018. 41488142 10.3389/fendo.2025.1694018 PMC12757215

[18] Heyer J Volmering S Hoyos-Banchon C Koc S Jeremiah L Steinseifer U . In vitro investigation of elevated hemolysis susceptibility in neonatal blood. Front Pediatr. (2025) 13:1616084. 40933703 10.3389/fped.2025.1616084 PMC12417390

